# Clinical characteristics of older and younger patients infected with SARS-CoV-2

**DOI:** 10.18632/aging.103535

**Published:** 2020-06-22

**Authors:** Zhiguo Zhou, Min Zhang, Yali Wang, Fang Zheng, Yaxiong Huang, Kang Huang, Qizhi Yu, Chunlin Cai, Dong Chen, Yi Tian, Jianhua Lei, Xinqiang Xiao, Erik De Clercq, Guangdi Li, Yuanlin Xie, Guozhong Gong

**Affiliations:** 1 The First Hospital of Changsha, Changsha, Hunan, China; 2 Institute of Hepatology and Department of Infectious Diseases, The Second Xiangya Hospital, Central South University, Changsha, Hunan, China; 3 Hunan Provincial Key Laboratory of Clinical Epidemiology, Xiangya School of Public Health, Central South University, Changsha, China; 4 Department of Microbiology, Immunology and Transplantation, Rega Institute for Medical Research, KU Leuven, Belgium

**Keywords:** SARS-CoV-2, COVID-19, older patients, C-reactive protein

## Abstract

Background: SARS-CoV-2 causes high mortality risk in older patients. This study aims to characterize the clinical features of older and younger SARS-CoV-2 infected patients.

Results: A total of 239 patients were divided into the younger group (<60 years; n=181) and the older group (≥60 years; n=58). In both groups, fever and cough were common symptoms. However, dyspnea was more frequent in older patients than younger patients (20.7% versus 9.9%, p=0.032). Compared with younger patients, older patients harbored more severe cases (37.9% versus 17.1%, p=0.001) and comorbidities (58.6% versus 21.0%, p<0.001) such as hypertension and diabetes. The baseline values of eosinophils and C-reactive protein were abnormal in older and younger groups. From baseline to day 14, significant decreases of three biomarkers (C-reactive protein, hemoglobin, albumin) and dramatic increases of three biomarkers (lymphocytes, platelets, blood urea nitrogen) were observed in older patients.

Conclusion: Older and younger patients exhibited differences in dyspnea, comorbidities, and proportions of severe cases. Moreover, the disease progression of SARS-CoV-2 in older patients is observed with the dynamics of laboratory biomarkers, supporting their potential use in disease monitoring.

Methods: We retrieved clinical symptoms, laboratory findings, comorbidities, and hospitalization information of SARS-CoV-2 cases in Changsha.

## INTRODUCTION

Severe acute respiratory syndrome coronavirus 2 (SARS-CoV-2) is a highly contagious coronavirus that causes pneumonia-like deaths and spreads fast through human-to-human contact [1–3]. Since the first suspected cases were documented in early December 2019, the increasing number of SARS-CoV-2 cases has reached more than six millions, including >350,000 deaths worldwide by June 1^th^, 2020.

It has been hypothesized that older people are more vulnerable to SARS-CoV-2. An early study reported a high prevalence of SARS-CoV-2 in older males with comorbidities [4]. Subsequent studies confirmed that SARS-CoV-2 was often observed in older patients with comorbidities [5] and severe disease progression [6, 7]. A study of 138 hospitalized patients reported a higher rate of ICU admission in older patients compared with younger patients [8]. Another study revealed a high risk of mortality in older patients with comorbidities and acute respiratory distress syndrome [9]. The overall case-fatality rate was 1.38% in China, but this rate increased to 3.99% in older patients between 60 and 69 years, 8.61% in older patients between 70 and 79 years, and 13.4% in ≥80 patients [10]. However, few studies have revealed clinical differences between older and younger groups.

Our study aims to characterize the clinical features of SARS-CoV-2 in younger and older groups based on a large cohort of 239 patients in Changsha - a neighboring city of Wuhan. Moreover, we assessed clinical symptoms, laboratory findings, comorbidities, and hospitalization information to monitor the disease progression of SARS-CoV-2.

## RESULTS

### Demographic profiles and clinical characteristics

**Figure 1:**
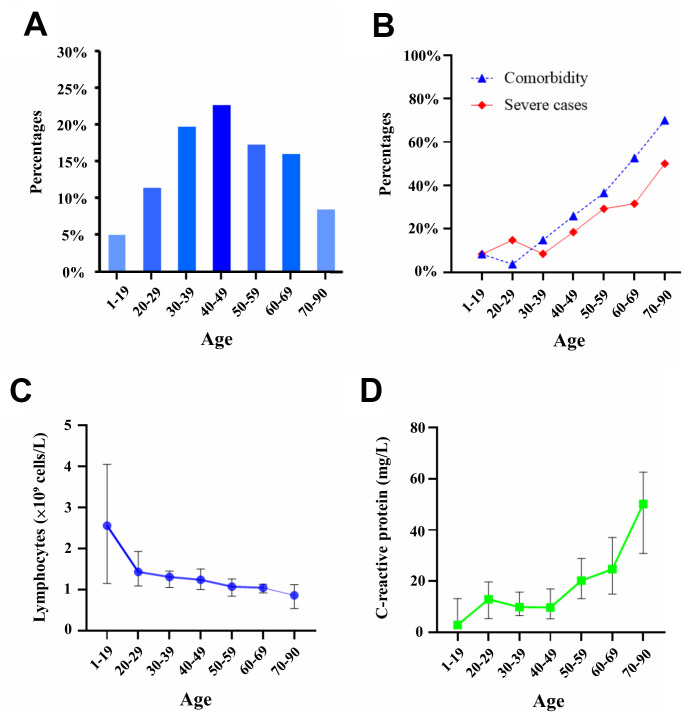
**Distribution of patient age and age-related biomarkers.** (**A**) Distribution of patients within decades of age. (**B**) Percentages of severe cases and patients with at least one comorbidity. (**C**) Serum levels of lymphocytes in seven age classes. (**D**) Serum levels of C-reactive protein in seven age classes.

A total of 239 patients confirmed with SARS-CoV-2 infections were hospitalized in Changsha. Table 1 summarizes their demographic and clinical characteristics. The median age of 239 patients was 45 years (interquartile range: 34 to 59 years) and 58 (24.3%) patients had at least 60 years of age (Figure 1A). Nearly half of the 239 patients were males. The youngest patient was a one-year-old girl discharged on February 18 after a 15-day hospitalization, while the eldest patient was an 84-year-old woman who had a 19-day hospitalization (from February 6 to February 25). Fifty-three (22.2%) patients were categorized into the severe group and severe cases were often observed in the elderly patients (Figure 1B).

**Table 1:** Clinical features of 239 patients infected with SARS-CoV-2.

	**Total (n=239)**	**Age<60 (n=181)**	**Age≥60 (n=58)**	**p-value**
**Age**	45.0(34.0-58.5)	40(31.0-47.0)	66.0(64.0-70.8)	<0.001
**Male**	118 (49.4%)	96 (53.0%)	22 (37.9%)	0.045
**Severe cases**	53 (22.2%)	31 (17.1%)	22 (37.9%)	0.001
**Comorbidity**				
Any	72 (30.1%)	38 (21.0%)	34 (58.6%)	<0.001
Hypertension	32 (13.4%)	11 (6.1%)	21 (36.2%)	<0.001
Diabetes	15 (6.3%)	6 (3.3%)	9 (15.5%)	0.001
Cardiovascular disease	10 (4.2%)	4 (2.2%)	6 (10.3%)	0.007
Hepatitis	7 (2.9%)	4 (2.2%)	3 (5.2%)	0.244
Chronic obstructive pulmonary disease	5 (2.1%)	3 (1.7%)	2 (3.4%)	0.407
Cerebral infarction	5 (2.1%)	2 (1.1%)	3 (5.2%)	0.060
Peptic ulcer	4 (1.7%)	4 (2.2%)	0 (0.0%)	0.254
Abnormal lipid metabolism	3 (1.3%)	1 (0.6%)	2 (3.4%)	0.085
Cardiac arrhythmia	3 (1.3%)	2 (1.1%)	1 (1.7%)	0.712
Chronic kidney disease	1 (0.4%)	1 (0.6%)	0 (0.0%)	0.571
**Symptoms**				
**Any**	222 (92.9%)	167 (92.3%)	55 (94.8%)	0.509
Fever	161 (67.4%)	124 (68.5%)	37 (63.8%)	0.505
Cough	139 (58.2%)	107 (59.1%)	32 (55.2%)	0.596
Fatigue	81 (33.9%)	56 (30.9%)	25 (43.1%)	0.089
Dyspnea	30 (12.6%)	18 (9.9%)	12 (20.7%)	0.032
Sore throat	27 (11.3%)	22 (12.2%)	5 (8.6%)	0.459
Myalgia	23 (9.6%)	17 (9.4%)	6 (10.3%)	0.830
Diarrhea	20 (8.4%)	14 (7.7%)	6 (10.3%)	0.532
Headache	18 (7.5%)	12 (6.6%)	6 (10.3%)	0.351
Dizziness	10 (4.2%)	7 (3.9%)	3 (5.2%)	0.666
Nausea or vomiting	8 (3.3%)	5 (2.8%)	3 (5.2%)	0.375
Runny nose	5 (2.1%)	3 (1.7%)	2 (3.4%)	0.407

At hospital admission, fever (67.4%) was the most common symptom, followed by cough (58.2%), fatigue (33.9%), dyspnea (12.6%), sore throat (11.3%), myalgia (9.6%), diarrhea (8.4%), and others (Table 1). In addition, 72 (30.1%) patients had at least one comorbidity such as hypertension (13.4%), diabetes (6.3%), cardiovascular disease (4.2%), hepatitis (2.9%), chronic obstructive pulmonary disease (2.1%), cerebral infarction (2.1%), peptic ulcer (1.7%), cardiac arrhythmia (1.3%), and abnormal lipid metabolism (1.3%). Furthermore, the increased risk of comorbidities was associated with the patient age that elderly patients were more likely to develop comorbidities (Figure 1B). HIV infection was absent in all patients.

### Older patients (≥60 years) and younger patients (<60 years)

Clinical features of 58 older patients and 181 younger patients were summarized in Table 1. The median ages of older and younger patients were 66 and 40 years, respectively (p<0.001). The percentage of females was higher in the older group than the younger group (62.1% versus 47.0%, p=0.045). Compared with younger patients, older patients had pronounced key features such as: (i) older patients were more likely to be severe (37.9% versus 17.1%, p=0.001); (ii) older patients harbored more comorbidities such as hypertension (36.2% versus 6.1%, p<0.001), diabetes (15.5% versus 3.3%, p=0.001), and cardiovascular disease (10.3% versus 2.2%, p=0.007); and (iii) older patients had more cases of dyspnea (20.7% versus 9.9%, p=0.032).

**Figure 2:**
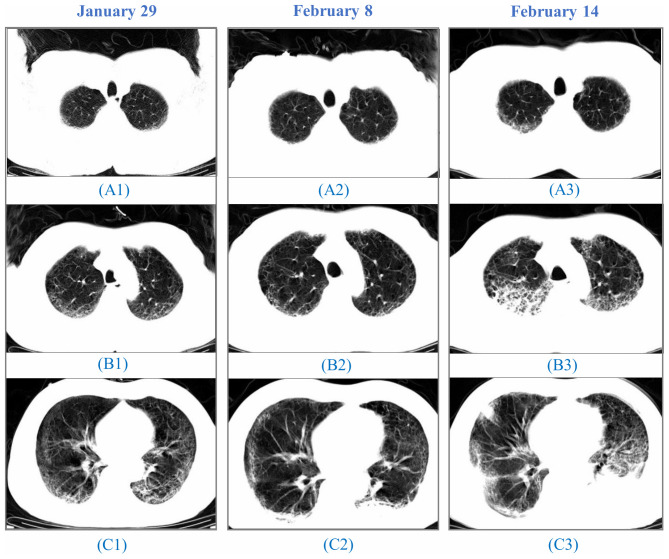
**CT images from a 64-year-old man.** A 64-year-old man, who had a fever and pneumonia, was suspected as the SARS-CoV-2 carrier on January 28 and confirmed on January 30. (**A1**), (**B1**) to (**C1**): On January 29, initial CT scans at the hospital admission showed multifocal ground-glass opacity (GGO) and reticulation, predominantly in the subpleural areas of both lungs. (**A2**), (**B2**) to (**C2**): On February 8, CT images indicated progressing GGOs. Newly-appeared patchy and core-like consolidation were visible in lower lobes of both lungs. The patient showed high fever, cough, blood in the sputum, reduced SpO2, and a sign of heart failure. (**A3**), (**B3**) to (**C3**): On February 14, CT images showed progressing lesion with multiple newly-appeared GGO and consolidation. Irregular interlobular septal thickening was observed in the upper lobe of the right lung. The patient passed away on February 15.

Comparisons of CT diagnostics in younger and older patients revealed no difference in the risk of abnormal lungs (p=0.972). Two males were severely ill during hospitalization and died thereafter. CT images showed the accumulation of ground-glass opacities and pulmonary consolidation during the disease progression (Figure 2).

### Biomarker dynamics during the disease progression of SARS-CoV-2

We compared laboratory biomarkers in older and younger patients at baseline (Table 2). The baseline values of eosinophils, C-reactive protein, lactic acid were abnormal in older and younger groups, while six biomarkers (white blood cells, neutrophils, alanine aminotransferase, total bilirubin, creatinine, lactic acids) were similarly expressed in both groups (Table 2). Of interest, the decrease of lymphocytes (Figure 1C) and the increase of C-reactive protein (Figure 1D) were observed along with the increasing age when patients were categorized based on their ages in decades (1 to 19, 20 to 29, 30 to 39, 40 to 49, 50 to 59, 60 to 69, ≥70 years).

**Table 2:** Baseline characteristics of biomarkers in 239 patients with SARS-CoV-2.

	**Total (n=239)**	**Age<60 (n=181)**	**Age≥60 (n=58)**	**p-value**
White blood cells (×10^9^ cells/L)	4.58(3.48-5.69)	4.59(3.47-5.73)	4.55(3.63-5.41)	0.523
Lymphocytes (×10^9^ cells/L)	1.14(0.85-1.60)	1.25(0.90-1.70)	0.98(0.64-1.19)	<0.001
Neutrophils (×10^9^ cells/L)	2.89(2.12-3.64)	2.86(2.03-3.61)	3.02(2.39-3.66)	0.261
Eosinophils (×10^9^ cells/L)	0.01(0-0.04)	0.01(0-0.05)	0.01(0-0.03)	0.010
Hemoglobin (g/L)	130(120-141)	132(122-143)	123(115.50-134.75)	0.001
Platelets (×10^9^/L)	171(138-227)	179(146-228)	147.50(117.25-206.50)	0.005
D-dimer (mg/L)	0.27(0.14-0.54)	0.22(0.13-0.48)	0.38(0.17-0.71)	0.013
C-reactive protein (mg/L)	15.60(4.36-30.85)	12.10(3.55-24.07)	30.11(15.80-55.19)	<0.001
Alanine aminotransferase (U/L)	19.45(14.21-27.48)	19.69(14.20-27.79)	18.22(14.22-26.36)	0.486
Aspartate aminotransferase (U/L)	24.40(19.80-31.37)	23.38(18.85-28.75)	27.90(23.46-35.87)	<0.001
Total bilirubin (μmol/L)	10.87(8.19-15.80)	10.92(8.02-15.60)	10.80(9.02-16.07)	0.665
Albumin (g/L)	38.23(35.36-40.97)	38.98(36.21-41.75)	35.56(32.17-38.32)	<0.001
Albumin/globulin	1.50(1.31-1.72)	1.53(1.39-1.78)	1.33(1.23-1.48)	<0.001
Blood urea nitrogen(mmol/L)	4.22(3.19-5.11)	4.12(3.14-4.88)	4.70(3.88-6.45)	0.003
Creatinine (μmol/L)	50.21(39.99-63.34)	49.85(39.8-62.19)	52.44(43.41-64.90)	0.227
Lactic acid	768.35(392.65-824.70)	761.40(391.60-808.10)	773.40(742.50-837.30)	0.482

Compared with younger patients, older patients had many abnormal biomarkers at baseline, including (i) higher levels of C-reactive protein (30.1 mg/L versus 12.1 mg/L, p<0.001); (ii) high levels of aspartate aminotransferase (27.90 U/L versus 23.38 U/L, p<0.001) and blood urea nitrogen (4.7 mmol/L versus 4.1 mmol/L, p=0.003); (iii) lower levels of hemoglobin (123 g/L versus 132 g/L, p=0.001) and albumin (35.56 g/L versus 38.98 g/L, p<0.001); and (iv) lower levels of lymphocytes (0.98 versus 1.25 ×10^9^ cells/L, p<0.001). The percentage of patients with lymphocytopenia was higher in older patients than younger patients (32.8% versus 17.7%, p=0.015), while normal leukocytes (range: 0.8 to 4×10^9^ cells/L) were observed in most patients (185, 77.4%).

**Figure 3:**
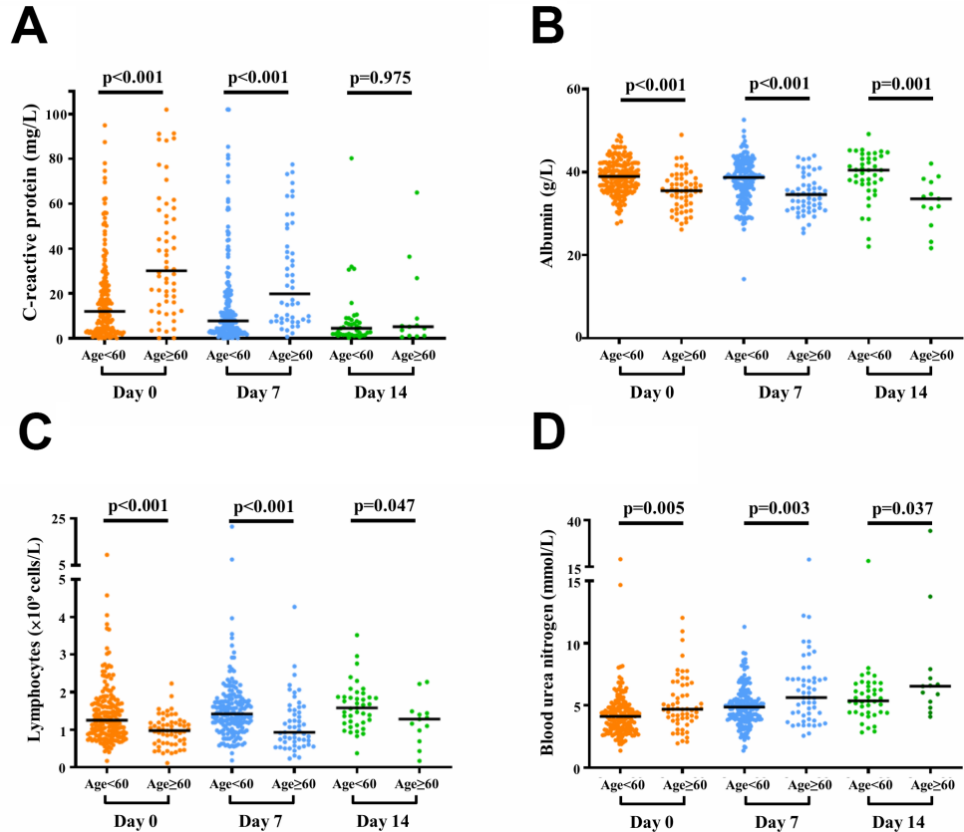
**Dynamics of laboratory biomarkers of 239 SARS-CoV-2 cases.** Scatter plots of C-reactive protein (**A**), albumin (**B**), lymphocytes (**C**), and blood urea nitrogen (**D**) in older and younger patients are illustrated on days 0, 7, and 14. Blood tests on days 0, 7, and 14 were conducted for 239, 229, and 54 patients, respectively. Laboratory biomarkers on day 14 were assessed for 54 patients who had positive SARS-CoV-2 and remained in hospital on day 14.

We next evaluated laboratory biomarkers of C-reactive protein, albumin, lymphocytes, and blood urea nitrogen on days 0, 7, and 14. (Figure 3). First, serum levels of C-reactive protein were higher (30.11 mg/L) in older patients at hospital admission, but it dropped sharply after treatment and returned to the normal status (5.2 mg/L) on day 14. In contrast, the lower level of C-reactive protein was observed in younger patients (12.1 mg/L) at baseline and it decreased slowly compared with that in older patients. Second, lymphocytes increased from 1.14×10^9^ cells/L at baseline to 1.34×10^9^ cells/L on day 7 and 1.46 ×10^9^ cells/L on day 14. Similar increasing patterns were observed for blood urea nitrogen from baseline (4.22 mmol/L) to day 14 (5.64 mmol/L). Third, serum levels of albumin in older patients continuously decreased from 35.56 g/L at baseline, to 34.6 g/L on day 7 and 33.6 g/L on day 14.

### Clinical outcome

**Figure 4:**
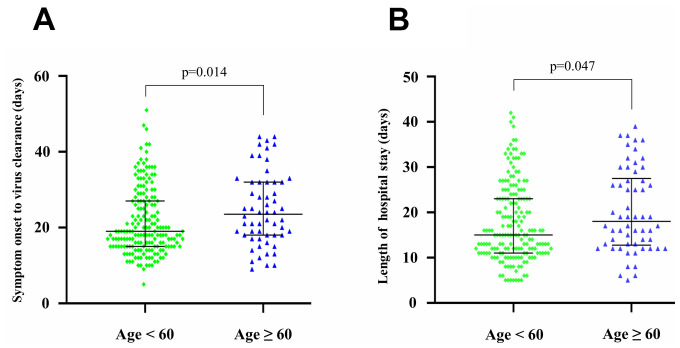
**Clinical features of 239 SARS-CoV-2 infected patients.** (**A**) The duration from symptom onset to virus clearance in younger and older patients. (**B**) The length of hospital stay in younger and older patients.

The median duration from symptom onset to virus clearance was 19 days (interquartile range: 15 to 28 days). This duration was much longer in older patients than younger patients (24 versus 19 days, p=0.014) (Figure 4A). Compared with younger patients, older patients had longer hospital stays (18 versus 15 days, p=0.047) (Figure 4B). We further analyzed the associations of baseline biomarkers with the short (<3 weeks) or long (≥3 weeks) hospital stay in older and younger patients (Supplementary Table 1). Blood urea nitrogen was significantly lower in older patients with a short hospital stay than older patients with a long hospital stay (p-value=0.037). Compared to older patients, younger patients with a short hospital stay usually had lower baseline levels of C-reactive protein and aspartate aminotransferase, but higher levels of lymphocytes, albumin, and albumin/globulin at baseline (p-values<0.05, Supplementary Table 1).

By March, 15^th^, 2020, 237 (99.2%) patients fulfilled the discharge criteria, and a 58-year-old male and a 64-year-old male had died. After the 14-day hospitalization, 129 patients were diagnosed with virus clearance, and 101 of them were discharged for 14-day home isolation. After hospital discharge, the presence of SARS-CoV-2 was not reported in any discharged patient over a follow-up period of two months.

## DISCUSSION

Based on a cohort of 239 patients, our study revealed three major findings: (i) older and younger patients exhibited differences in dyspnea, comorbidities, and proportions of severe cases; (ii) compared with younger patients, older patients exhibited higher levels of C-reactive protein, D-dimer, aspartate aminotransferase, blood urea nitrogen and lower levels of lymphocytes, hemoglobin, platelet, albumin at baseline; and (iii) the disease progression of SARS-CoV-2 was associated with the dynamics of laboratory biomarkers such as C-reactive protein and lymphocytes, supporting their clinical use in disease monitoring.

SARS-CoV-2 is a highly pathogenic coronavirus of bat origin [11] that causes upper respiratory tract diseases and pneumonia-like diseases [3, 9]. Increased mortality risk was previously reported in critically ill patients with chronic comorbidities and acute respiratory distress syndrome [9]. Similar to the prevalence of older patients in Wuhan [4], 58 (24.3%) of 239 patients were ≥60 years in our study. Although the cutoff of 60 years was used to categorize older and younger patients, key factors such as comorbidities, severe cases, lymphocytes, and C-reactive protein showed increasing or decreasing patterns over seven age groups (Figure 1). In agreement with previous studies [5], severe cases were often observed in older patients in our study (Table 1). Older patients often have many comorbidities such as diabetes, hypertension, and cardiovascular disease, which potentially cause the difficulty of clinical treatment. Fever, cough, and fatigue were common symptoms but there were no differences in older and younger patients, indicating that symptoms and signs were not unique features to distinguish the impact of SARS-CoV-2 in both groups. However, dyspnea was more common in older patients, implying the potential risk of lung lesions after SARS-CoV-2 infection.

In agreement with previous studies [6, 12, 13], our study revealed key laboratory markers such as white blood cells, lymphocytes, eosinophils, C-reactive protein, albumin, blood urea nitrogen, aspartate aminotransferase, and lactic acid. These biomarkers are commonly used to monitor disease progression, inflammatory/immune responses, and/or physiological changes associated with viral infections. For instance, lymphocytes are a type of white blood cell that could play protection roles in defending viral infections [14]. Lymphocytopenia might be a biomarker to reveal disease severity or antiviral immunity [15]. Lymphocytopenia was observed in 51 (21.3%) patients in our study, while this percentage was lower than the national study (83.2%) [3]. This discordance may be due to the condition of mildly ill patients in our cohort and timely antiviral treatment. Moreover, C-reactive protein was much higher in older patients (30.11 mg/L versus 12.11 mg/L), indicating that severe inflammatory reactions could be observed in older patients. Whether these biomarkers could be effective predictors of treatment responses requires further investigations.

This study has several limitations. First, our study characterized older and younger patients in Changsha, but the clinical features of older patients should be analyzed from a global perspective, including those from different countries. Second, we evaluated the dynamics of laboratory biomarkers in a 14-day period because of data availability, but future studies should report the full course of disease progression. Third, only two deaths were observed in our study and future studies should characterize the mortality risk of older patients in larger cohorts.

## CONCLUSION

Overall, our study characterized the clinical features of younger and older patients infected with SARS-CoV-2. Older and younger patients exhibited differences in dyspnea, comorbidities, and proportions of severe cases. Higher levels of C-reactive protein, aspartate aminotransferase, blood urea nitrogen, and lower levels of lymphocytes and albumin were observed in older patients. Furthermore, the dynamics of laboratory biomarkers such as lymphocytes and C-reactive protein can be used for monitoring the disease progression in older patients.

## MATERIALS AND METHODS

### Study design and participants

This study was conducted at The First Hospital of Changsha, designated as the single hospital to treat all SARS-CoV-2 cases in Changsha. Patients who were infected with laboratory-confirmed SARS-CoV-2 according to the WHO interim guidance [16] were transferred from local hospitals to The First Hospital of Changsha between January 23 and March 15, 2020. Clinical outcomes were monitored up to the hospital discharge of all patients. This study was performed following the Helsinki Declaration and was approved by the Ethics Committee of The First Hospital of Changsha. In light of the rapid emergence of SARS-CoV-2, written informed consent was waived for this observational study. The corresponding author had full access to all the data in the study and had final responsibility for the decision to submit for publication.

### Data collection

We retrieved electronic medical records, clinical symptoms or signs, laboratory findings, comorbidities, and hospitalization information of hospitalized patients infected with SARS-CoV-2. Clinical information was retrieved using a customized collection form. Any missing or uncertain record was clarified by direct communications with doctors and patients. To verify data accuracy, two study investigators (HYX and CCL) reviewed the clinical data independently.

### Diagnostics of SARS-CoV-2

To identify the presence of SARS-CoV-2, throat swab specimens were collected for real-time RT-PCR analyses using the SLAN-96P real-time PCR system (Hongshitech, Shanghai, China) and SARS-CoV-2 nucleic acid diagnostic kits (PCR-Fluorescent Probe) from Sansure Biotech, Changsha, China. The latter was approved by the China National Medical Products Administration (registration number: 20203400064) and the European CE approval (ID: CMB 8764-2020). The detection limit of this nucleic acid kit was 200 copies/mL. SARS-CoV-2 tests were independently conducted at two medical centers: the First Hospital of Changsha and the Changsha Municipal Center for Disease Prevention and Control. A positive case was reported if SARS-CoV-2 was identified by two medical centers above, while a negative case was reported if two medical centers consistently reported an undetectable viral load. Negative cases were considered from their discharge if they fulfilled three requirements: (i) no respiratory symptoms of fever or cough were observed for three consecutive days; (ii) two consecutive nucleic acid tests were negative (three days apart from each test); and (iii) computed tomography images became normal. All discharged cases remained on home isolation for another 14 days.

### Laboratory assessments

Computed tomography (CT) diagnostics were performed using the 128-slice SOMATOM go. Top CT systems from Siemens Healthineers. Hematologic assessments of white blood cells, hemoglobin, lymphocytes, neutrophils, eosinophils, and platelets were proceeded using the Mindray BC-6800 automated hematology analyzer. Biochemical data of albumin, alanine aminotransferase, albumin/globulin, aspartate aminotransferase, blood urea nitrogen, creatinine, C-reactive protein, D-dimer, and total bilirubin were quantified using the ARCHITECT c16000 clinical chemistry analyzer.

### Classification of severe and non-severe cases

Based on the New Coronavirus Diagnosis and Treatment Guideline (version 7) in China, a severe case was classified if a patient had any of the following conditions: (i) respiratory distress with the respiration rate ≥30 times per minute; (ii) oxygen saturation ≤93% in the resting state; (iii) the ratio of the arterial partial pressure of oxygen to fraction of inspired oxygen ≤300 mmHg (1mmHg = 0.133 kPa); and (iv) the area of the lung affected with pneumonia increased >50% within 24 to 48 hours. Non-severe cases included mild or moderate patients who had the conditions of fever (≥37.5°C) and/or the respiratory tract.

### Statistical analyses

We measured median (interquartile range) of continuous variables as well as counts and percentages of categorical variables. Normal distribution was examined by Shapiro-Wilks normality tests. To explore differences between patient groups, the chi-square and Fisher’s exact tests were conducted for categorical variables; two-tailed t-tests were performed for continuous variables following normal distributions; the Wilcoxon rank-sum tests were used for non-normal continuous variables in paired groups; Mann-Whitney U tests were applied for non-normal continuous variables in unpaired groups. A common approach called pairwise deletion was applied to handle missing data. Statistical analyses were conducted using SPSS 16.0. Differences were considered significant at p<0.05.

## Supplementary Material

Supplementary Table 1
