# Supplementary material for: Clinical characteristics of older and younger patients infected with SARS-CoV-2
Source: Aging (Albany NY). 2020 Jun 22;12(12):11296–305. doi: 10.18632/aging.103535 (PMC7343458; doi:10.18632/aging.103535)
Supplement: Supplementary Table 1 [file aging-12-103535-s001..pdf]

SUPPLEMENTARY TABLE

Supplementary Table

Supplementary Table 1. Baseline features of laboratory biomarkers in older and younger patients.

Biomarkers	Group 1 (G1) Younger patients Hospital stay < 21 d (N=122)	Group 2 (G2) Younger patients Hospital stay ≥21 d (N=59)	G1 vs G2 p-value	Group 3 (G3) Older patients Hospital stay < 21d (N=35)	Group 4 (G4) Older patients Hospital stay ≥21d (N=23)	G3 vs G4 p-value	G1 vs G3 p-value	G1 vs G4 p-value	G2 vs G3 p-value	G2 vs G4 p-value
White blood cells	4.67(3.49-5.90)	4.53(3.41-5.61)	0.299	4.94(3.81-5.53)	4.10(3.12-5.03)	0.129	0.871	0.123	0.356	0.366
Lymphocytes	1.35(0.93-1.79)	1.15(0.84-1.61)	0.094	1.05(0.86-1.21)	0.74(0.63-1.14)	0.133	**0.002**	**<0.001**	**0.045**	**0.003**
Neutrophils	2.85(2.05-3.66)	2.90(2.05-3.50)	0.675	3.08(2.52-3.85)	2.74(2.00-3.26)	0.215	0.131	0.764	0.081	0.918
Eosinophils	0.02(0.01-0.07)	0.01(0.00-0.04)	0.053	0.01(0.00-0.03)	0.01(0.00-0.03)	0.150	0.058	0.002	0.981	0.097
Hemoglobin	132(120-142)	134 (124-145)	0.291	120 (115 -132)	125 (118-143)	0.224	0.001	0.428	<0.001	0.141
Platelet	176(144-227)	179 (152-234)	0.507	150 (134-213)	138(112-187)	0.192	0.172	0.011	0.075	0.006
D-dimer	0.21(0.12-0.52)	0.28(0.16-0.45)	0.393	0.38(0.18-0.85)	0.36(0.16-0.57)	0.338	0.009	0.257	0.053	0.535
C-reactive protein	10.30(4.14-20.29)	13.05(3.29-25.14)	0.443	24.90(14.97-44.20)	43.30(20.23-62.12)	0.141	**0.001**	**<0.001**	**<0.001**	**<0.001**
Alanine aminotransferase	19.45(13.75-27.21)	19.91(15.12-28.31)	0.666	19.70(14.86-28.00)	16.29(13.76-20.82)	0.117	0.669	0.148	0.941	0.093
Aspartate aminotransferase	23.23(18.87-30.13)	23.74(19.11-28.53)	0.712	27.50(22.87-34.71)	28.29(24.78-38.67)	0.431	**0.005**	**0.005**	**0.042**	**0.022**
Total bilirubin	10.92(8.05-15.32)	10.78(7.99-16.86)	0.605	10.72(8.89-17.37)	10.87(9.42-14.20)	0.691	0.483	0.963	0.639	0.84
Albumin	38.86(36.26-41.40)	39.41(36.17-42.17)	0.637	35.46(32.69-38.22)	36.0(31.22-38.3)	0.886	**<0.001**	**0.003**	**<0.001**	**0.003**
Albumin/globulin	1.52(1.39-1.79)	1.55(1.39-1.74)	0.951	1.34(1.21-1.57)	1.30(1.24-1.41)	0.499	**0.001**	**<0.001**	**0.002**	**<0.001**
Blood urea nitrogen	4.01(3.12-4.83)	4.29(3.20-5.05)	0.273	4.47(3.50-5.12)	5.23(4.14-7.07)	**0.037**	0.133	<0.001	0.647	0.007
Creatinine	48.81(38.43-58.91)	53.13(41.93-64.33)	0.092	51.45(43.11-63.08)	57.0(44.04-66.07)	0.413	0.320	0.105	0.719	0.512
Lactic acid	768.35(397-845)	742(387-789)	0.373	798(636-830)	769(742-841)	1.000	0.887	0.541	0.516	0.416
